# Mass of components and material distribution in lateral flow assay kits

**DOI:** 10.2471/BLT.24.292167

**Published:** 2025-02-04

**Authors:** Marie-Louise Wöhrle, Alice Street, Maïwenn Kersaudy-Kerhoas

**Affiliations:** a School of Social and Political Science, University of Edinburgh, Edinburgh, Scotland.; b School of Engineering and Physical Sciences, Heriot-Watt University, Edinburgh, EH14 4AS, Scotland.

## Abstract

**Objective:**

To assess the type and amount of materials used in commercial lateral flow tests.

**Methods:**

We collected and weighed the components of 21 commercial coronavirus disease 2019 (COVID-19) lateral flow tests from the European Union, the United Kingdom of Great Britain and Northern Ireland, the United States of America and the World Health Organization’s emergency use listing procedure. We took test kits apart manually, classified components and weighed them individually.

**Findings:**

We found large variations in the total average weights of the lateral flow kits ranging from 13.7 g per test to 84.6 g. The average weight of standard housing in the kits was 4.1 g per casing (range: 2.8–6.5). The packaging made up between 34% and more than 89% of the whole kit and was found to be a large source of weight variations. In the standard kits, plastics made up on average 36% of the total weight, while paper and cardboard accounted for 52% on average. In the non-standard kits, which had newer cassette designs, the opposite was observed.

**Conclusion:**

Wide variation in the weight of components in COVID-19 tests suggests there is scope for manufacturers to reduce the amount of materials, including plastic, in these products. We propose that a quantitative baseline of material usage be introduced in target product profiles for lateral flow tests to limit the large volume of plastic from reaching the market, and reduce the burden of plastic waste from diagnostic testing on local waste management systems.

## Résumé

**Objectif:**

Évaluer le type et la quantité de matières utilisées dans les tests commerciaux à flux latéral.

**Méthodes:**

Nous avons collecté et pesé les composants de 21 tests commerciaux à flux latéral employés dans la détection de la maladie à coronavirus 2019 (COVID-19). Ces tests provenaient de l’Union européenne, du Royaume-Uni de Grande-Bretagne et d’Irlande du Nord, des États-Unis d’Amérique et de la procédure d’inscription sur la liste des utilisations d’urgence de l’Organisation mondiale de la santé. Nous avons démonté manuellement les kits de dosage, en avons classé les composants et les avons pesés individuellement.

**Résultats:**

Nous avons constaté de grandes variations dans le poids total moyen des kits de dosage à flux latéral: de 13,7 g à 84,6 g par test. Le poids moyen des logements standard dans les kits était de 4,1 g (plage: 2,8–6,5). Le conditionnement représentait entre 34% et plus de 89% de l’ensemble du kit et s’est avéré être une source importante de variation du poids. Dans les kits standard, les matières plastiques représentaient en moyenne 36% du poids total, tandis que le papier et le carton en représentaient 52% en moyenne. Dans les kits non standard, qui comportaient des cassettes de conception plus récente, c’est l’inverse qui a été observé.

**Conclusion:**

La grande variation du poids des composants dans les tests COVID-19 suggère que les fabricants ont la possibilité de réduire la quantité de matières, y compris les matières plastiques, dans ces produits. Nous proposons d’introduire une référence quantitative pour l’utilisation des matières dans les profils de produits cibles pour les tests à flux latéral afin de limiter l’arrivée sur le marché d’un volume important de matières plastiques et de réduire la charge des déchets plastiques issus des tests de diagnostic sur les systèmes locaux de gestion des déchets.

## Resumen

**Objetivo:**

Evaluar el tipo y la cantidad de materiales utilizados en las pruebas comerciales de flujo lateral.

**Métodos:**

Se recogieron y pesaron los componentes de 21 pruebas comerciales de flujo lateral de la enfermedad por coronavirus de 2019 (COVID-19) de los Estados Unidos de América, el Reino Unido de Gran Bretaña e Irlanda del Norte, la Unión Europea y el procedimiento de inclusión en la lista de uso de emergencia de la Organización Mundial de la Salud. Se desmontaron manualmente los kits de pruebas, se clasificaron los componentes y se pesaron por separado.

**Resultados:**

Se encontraron grandes variaciones en los pesos medios totales de los kits de flujo lateral, que oscilaron entre 13,7 g por prueba y 84,6 g. El peso medio de la envoltura estándar de los kits fue de 4,1 g por envoltura (intervalo: 2,8-6,5). El envase representaba entre el 34% y más del 89% de todo el kit y resultó ser una fuente importante de variaciones de peso. En los kits estándar, los plásticos representaban una media del 36% del peso total, mientras que el papel y el cartón suponían una media del 52%. En los kits no estándar, que tenían diseños de casete más nuevos, se observó lo contrario.

**Conclusión:**

La gran variación en el peso de los componentes en las pruebas de la COVID-19 sugiere que hay posibilidades de que los fabricantes reduzcan la cantidad de materiales, incluido el plástico, en estos productos. Se propone la introducción de una referencia cuantitativa del uso de materiales en los perfiles de los productos objetivo de las pruebas de flujo lateral para limitar el gran volumen de plástico que llega al mercado y reducir la carga que suponen los residuos plásticos de las pruebas de diagnóstico para los sistemas locales de gestión de residuos.

## ملخص

الغرض تقييم نوع وكمية المواد المستخدمة في اختبارات التدفق الجانبي التجارية.

الطريقة قمنا بجمع مكونات 21 اختبارًا للتدفق الجانبي التجاري لمرض فيروس كورونا 2019 (كوفيد 19) والقيام بوزنها، وكانت هذه الاختبارات من الاتحاد الأوروبي، والمملكة المتحدة لبريطانيا العظمى وأيرلندا الشمالية، والولايات المتحدة الأمريكية، فضلاً عن إجراء قائمة الاستخدام الطارئ لمنظمة الصحة العالمية. قمنا بفصل مجموعات الاختبار يدويًا، وتصنيف المكونات، ووزنها بشكل فردي.

النتائج وجدنا تنوعات كبيرة في متوسط الأوزان الإجمالية لمجموعات التدفق الجانبي، تتراوح من 13.7 جرامًا إلى 84.6 جرامًا لكل اختبار. كان متوسط وزن الغلاف القياسي في المجموعات 4.1 جرامًا لكل غلاف (النطاق: 2.8 إلى 6.5). شكلت العبوة ما بين %34 وأكثر من %89 من المجموعة بأكملها، وتبين أنها مصدر كبير لتنوعات الوزن. في مجمعات الاختبار القياسية، شكّلت المواد البلاستيكية في المتوسط %36 من الوزن الإجمالي، بينما شكّل الورق والكرتون %52 في المتوسط. وفي مجموعات الاختبار غير القياسية، التي كانت تحتوي على تصميمات حاويات أحدث، تمت ملاحظة العكس.

الاستنتاج تشير التنوعات الكبيرة في وزن المكونات في اختبارات كوفيد 19 إلى وجود مجال أمام الشركات المصنعة لتقليل كمية المواد في هذه المنتجات، بما في ذلك البلاستيك. نحن نقترح تقديم خط أساس كمي لاستخدام المواد في ملفات تعريف المنتجات المستهدفة لاختبارات التدفق الجانبي، بهدف الحد من الحجم الكبير للبلاستيك من الوصول إلى السوق، وتقليل عبء النفايات البلاستيكية من الاختبارات التشخيصية على أنظمة إدارة النفايات المحلية.

## 摘要

**目的:**

评估商用侧流检测装置中所用材料的类型和数量。

**方法:**

我们收集了符合欧盟、大不列颠及北爱尔兰联合王国、美利坚合众国和世界卫生组织紧急使用清单程序标准的 21 种商用 2019 冠状病毒病 (COVID-19) 侧流检测装置并对其组件进行了称重。我们手动拆开检测试剂盒，对组件进行分类，并分别称重。

**结果:**

我们发现侧流检测试剂盒的平均总重量差别很大，每份检测材料从 13.7 克到 84.6 克不等。标准试剂盒外壳的平均重量为每个盒子 4.1 克（范围：2.8-6.5 克）。包装重量占整个试剂盒重量的 34% 至 89% 以上，这是导致试剂盒重量存在变化的主要原因。在标准试剂盒中，塑料平均占总重量的 36%，而纸质材料和纸板平均占总重量的 52%。在采用较新卡式设计的非标准试剂盒中，我们发现情况刚好相反。

**结论:**

COVID-19 检测装置中组件重量变化幅度很大的现象表明，制造商还可努力减少这些产品中材料（包括塑料）的用量。我们建议在侧流检测相关目标产品简介中给出所用材料定量基线，以限制大量塑料进入市场，并减少当地废物管理系统在处理因诊断检测而产生的塑料废物方面的负担。

## Резюме

**Цель:**

Оценить тип и количество материалов, используемых при проведении коммерческих ИХА на тест-полосках.

**Методы:**

Были собраны и взвешены компоненты 21 коммерческого набора для ИХА на тест-полосках, которые использовались для выявления коронавирусной болезни 2019 года (COVID-19) в странах Евросоюза, Соединенного Королевства Великобритании и Северной Ирландии, Соединенных Штатов Америки, а также упомянуты в инструкции по составлению перечня продукции, допущенной к применению в условиях чрезвычайной ситуации (ВОЗ). Наборы реагентов разбирались вручную, компоненты были классифицированы и взвешены по отдельности.

**Результаты:**

В среднем общая средняя масса наборов для выполнения ИХА на тест-полосках варьировалась от 13,7 г на тест до 84,6 г. Средняя масса стандартного корпуса в наборах составляла 4,1 г (диапазон: 2,8–6,5). Упаковка занимала от 34% до более чем 89% всего набора и была признана одним из основных источников колебаний массы. В стандартных наборах пластик составлял в среднем 36% от общей массы, бумага и картон – 52%. В нестандартных наборах с более современными конструкциями кассет наблюдалась обратная картина.

**Вывод:**

Значительные отклонения в массе компонентов тестов на COVID-19 свидетельствуют о том, что у производителей есть возможность сократить количество материалов, включая пластик, в этих продуктах. Для ограничения попадания на рынок большого количества пластика и снижения нагрузки на местные системы управления отходами предлагается ввести количественные показатели использования материалов в целевых профилях продуктов для выполнения ИХА на тест-полосках.

## Introduction

Rapid testing has become a pillar of global health, aiding responses to emerging disease outbreaks,^1^ expanding universal health coverage,^2^ tackling antimicrobial resistance^3^ and contributing to efforts to eliminate neglected diseases.^4^ In 2023, the Global Fund to Fight AIDS, Tuberculosis and Malaria invested in 53 million tests for human immunodeficiency virus and 321 million malaria tests. The annual production of lateral flow tests is reported to exceed 2 billion annually.^5^ A growing percentage of such tests are designed to be used in primary health-care settings or at home. These tests dispense with the need for expensive laboratory infrastructure and expertise. The fourth *World Health Organization* *Model list of essential in vitro diagnostics* includes over 35 tests for use in community settings or health facilities without laboratories, more than half of which are lateral flow tests.^6^

Lateral flow test kits are generally designed for single use. Over the past 60 years, single-use medical devices and materials, such as syringes, gloves, masks and diagnostic kits, have become ubiquitous in health systems globally. This reliance on single-use products has led to a substantial increase in medical waste, which contributes to global carbon emissions and local pollution.^7^^,^^8^ In settings lacking waste management infrastructure and regulation, waste from lateral flow testing kits and other single-use medical products can end up on beaches, in waterways, in municipal landfill or being openly burned.^9^ Even in settings with established waste management systems, lateral flow tests are not routinely recycled and their waste is only recovered as heat from incineration. The increased production and use of single-use plastic lateral flow cassettes is leading to considerable waste production and puts environmental health at risk.^9^ The amounts and types of materials used in such tests have never been rigorously investigated.

Factors driving the rise of single-use plastics in health care include convenience, savings on cleaning and sterilization, labour costs and suitability for mass production.^10^^,^^11^ The common rationale for the single-use design in medical devices is that it ensures sterile products, reduces the risk of cross-contamination, and protects patients and health workers from infectious diseases. However, growing evidence highlights examples of devices, such as drug and instrument trays^12^ and surgical drapes,^13^ where single-use design is not essential for infection control and patient safety. In other cases, opportunities have been identified for reducing product packaging,^14^ using biodegradable polymers in device manufacture^15^ and incorporating devices into recycling schemes,^16^^,^^17^ without a detrimental effect on medical efficacy or safety. These findings suggest that there is potential for innovations in the design and materials of single-use medical devices to tackle the environmental impact of health care without compromising safety standards.^18^^,^^19^

In 2019, experts reviewed progress in the development of accurate, accessible and affordable rapid diagnostics tests and recommended that so-called environmental friendliness be added to the World Health Organization (WHO)-endorsed, affordable, sensitive, specific, user-friendly, rapid and robust, equipment-free and deliverable (ASSURED) criteria for assessing such devices. The environmental considerations of the new so-called REASSURED criteria are limited to advising that “Completed tests are easy to dispose and manufactured from recyclable materials.”^20^ Considerable scope exists for these recommendations to be expanded to include opportunities for plastic reduction and substitution of materials at the design stage.

Target product profiles (that is, planning tools that guide development towards desired characteristics) are the best available tool for implementing criteria for sustainability. However, the environmental impact and waste disposal within target product profiles have not yet been considered. Global health organizations increasingly use target product profiles as a tool to establish consensus among stakeholders about priorities for test design and to communicate those needs and expectations to industry.^21^^,^^22^ By laying out both minimum and optimal requirements for test specifications, target product profiles balance existing technical capacities with future aspirations to stimulate innovation. They therefore offer an important point of intervention for establishing norms and standards for sustainable design in the diagnostics sector. While target product profiles generally define a broad range of characteristics on clinical need, analytical performance, clinical validity, infrastructure and human factors, and costs, environmental impact is less regularly considered in the development of these documents.^3^ In an expert consensus from 2015 to 2016, waste disposal was assigned low priority for detailed discussion by experts in a panel discussion in the shaping of a target product profile related to antimicrobial resistance.^23^

In this study, we aimed to assess the weights of components in commercial lateral flow tests for coronavirus disease 2019 (COVID-19) which could provide a base for establishing guidance on maximum quantitative weight thresholds for materials in future target product profiles for lateral flow tests.

## Methods

We collected 21 different COVID-19 lateral flow test kits, with emergency use authorizations from the Medicines and Healthcare Products Regulatory Agency of the United Kingdom of Great Britain and Northern Ireland, the European Union (EU), the Food and Drug Administration of the United States of America (USA), and the WHO emergency use listing procedure (Table 1). Despite concerted efforts, it was easier to obtain tests available for personal use that had market authorization in the United Kingdom than other tests. We were unable to obtain many tests from the WHO emergency use listing as these tests were only available in large quantities from manufacturers and resellers: Artron, Wondfo and Flowflex tests for professional use had a minimum order range of 800–1000. The Wondfo and Flowflex self-tests available in the United Kingdom do not have the same serial numbers as the WHO emergency use tests, but appear to originate from the same product line. We purchased 11 tests in the United Kingdom, six in France and Germany and another four in the USA.

**Table 1:** COVID-19 lateral flow tests kits collected

Test name	CE code	Kit reference code	Lot number	Country of purchase	No. of tests per kit	No. of individual tests weighed
Everything Genetic one-step-test for SARS-CoV-2 antigen	CE1434	CG20615	LOTPSC225172W	United Kingdom	1	3
StepAhead one-step-test for SARS-CoV-2 antigen	CE1434	CG20615	PSC220038W	United Kingdom	1	2
Fluorecare SARS-CoV-2 antigen test kit	CE1434	MF-68	NA	France	1	1
Medicspot lateral flow antigen travel service	CE0123	NA	COV1110005	United Kingdom	1	3
Flowflex SARS-CoV-2 antigen rapid test	CE0123	L031–118P5	COV2065033	United Kingdom	5	3
Orient Gene’s rapid COVID-19 (antigen) self-test	CE0123	GCCoV-502a-H70GE	2 201 750	United Kingdom	7	3
Hughes SARS-CoV-2 antigen rapid test (travel)	CE0123	REF L031–118M3E	COV1070067	United Kingdom	1	1
Getein one-step-test for SARS-CoV-2 antigen	CE1434	REF CG206155	PSC220040W	United Kingdom	5	3
Wondfo 2019-nCoV antigen test	CE0123	W634P0024	W63410603	United Kingdom	1	3
Wondfo 2019-nCoV antigen test	CE0123	W364P0028	W63410604	United Kingdom	2	3
Boson rapid SARS-CoV-2 antigen test card	CE0123	1N40C5–4	220 112 091	United Kingdom	5	3
Panbio COVID-19 Ag RTD nasopharyngeal	NA	41FK10	41ADH256A	Germany	25	3
BinaxNow COVID-19 antigen self-test	NA	REF 195–160	213 253	USA	2	3
iHealth COVID-19 antigen rapid test	NA	GTIN 20856362005894	221CO20130	USA	2	2
QuickVue at-home OTC COVID-19 test	NA	20 402	F41691	USA	2	2
Ecotest COVID-19 antigen nasal test kit	CE1434	COV-535002H5	I2112213	Germany	5	3
NHS test & trace COVID-19 self-test (RAT)	NA	TK2193	X2201712	United Kingdom	7	3
Hotgen coronavirus (2019-nCov) antigen test	CE0123	HGCG134S0101	W2022030400	Germany	1	1
Safecare COVID-19 antigen RTK	CE1434	COV Ag-6012H	COV22072001	Germany	1	1
NEWGENE COVID-19 antigen detection kit	CE1434	COVID-19-NG21	20 220 718–01	Germany	1	1
Ellume COVID-19 home test	NA	I-SRS-C-01	QB02S-H	USA	1	1

CE: Conformité Européenne; COVID-19: coronavirus disease 2019; NA: not applicable; NHS: National Health Service; OTC: over-the-counter; RAT: rapid antigen test; RTD: rapid test device; RTK: rapid test kit; SARS-CoV-2: severe acute respiratory syndrome coronavirus 2.

We took the tests apart manually: first we simply opened all the packaging. We used a scalpel to disassemble the lateral flow cassettes and extract the nitrocellulose strip or other similarly integrated components. A photograph of the dismantling process is in the online repository.^24^ We considered an individual component as one that cannot be further taken apart, for example, because it is made of a single block of plastics. These individual components were weighed using a Fisherbrand PS-60 (Fisherbrand, Hampton, USA) precision scale for weights lower than 60 g, and an Ohaus CL (Ohaus, Parsippany, USA) series scale for weights more than 60 g. We created categories and subcategories of components of interest. While the standard deviation (SD) between identical tests or components was negligible, typically less than 1%, nevertheless, where possible, we weighed at least three identical tests and averaged the total weight and individual component weights. For boxes with multiple tests, we calculated the weight of a single test by dividing the total of shared component weights by the number of tests. The content of each individual kit in the collection is shown in the online repository.^24^

We categorized the material of each component as plastics, paper or cardboard, as other if the material was metal or was a mix of components of different nature if the component could not be taken apart, or as unidentified.

We used descriptive statistics to describe the results, specifically, mean weights  and SD.

## Results

### Components of tests

Box 1 shows all identified test components and subcomponents. Briefly, all test kits contained a card or plastic-based outer packaging, instructions, swab pack, cassette pack and reagent pack. The swab pack typically contained a swab to obtain nasal or nasopharyngeal samples, which was packed in sterile paper and plastic packaging. The cassette packs usually consisted of an aluminium foil pouch containing a nitrocellulose strip encased in a hard plastic cassette, and a desiccant packet. The reagent pack generally had a prefilled reagent tube sealed with a foil and a cap. Beyond these key components, some kits included additional devices, such as a test-tube holder or rack, when the outer packaging itself did not serve as a test-tube holder. Many, but not all kits, included one biohazard or waste zip-lock bag per test to gather all used test components for disposal, or a certification paper slip. Some test kits also contained additional interior packaging such as large soft plastic zip-lock bags.

Box 1 **Component categories and subcategories of COVID-19 lateral flow test kits**

**A. Outer packaging**

A.1 Box

A.2 Outer foil pouch

A.3 Plastic wrapping

A.4 Additional cardboard packaging

**B. Instructions**

B.1 Instructions for use

B.2 Additional information

**C. Certification slip**

**D. Biosafety/waste bag**

**E. Swab pack**

E.1 Swab

E.2 Packaging part 1 (paper)

E.3 Packaging part 2 (plastic)

**F. Reagent pack**

F.1 Tube

F.2 Tube foil lid

F.3 Tube plug

F.4 Dropper (if separate from tube)

F.5 Screw-on cap

F.6 Blister pack

F.7 Reagent

F.8 Additional bags and/or packaging

F.9 Additional bags/packaging (foil)

F.10 Additional bags/packaging (rubber band)

F.11 Dessicant

F.12. Reagent bottle

**G. Cassette pack**

G.1 Packaging

G.2 Dessicant

G.3 Test cassette

G.3.1 Lateral flow strip

G.3.2 Outer casing top

G.3.3 Outer casing bottom

G.3.4 Battery

G.3.5 On/off button

G.3.6 Plastic lateral flow assay strip tray top

G.3.7 Plastic lateral flow assay strip tray bottom

G.3.8 Analyser printed circuit board

**H. Tube rack**

**I. Control swabs**

COVID-19: coronavirus disease 2019.

### Weight of the tests

**Fig. 1:**
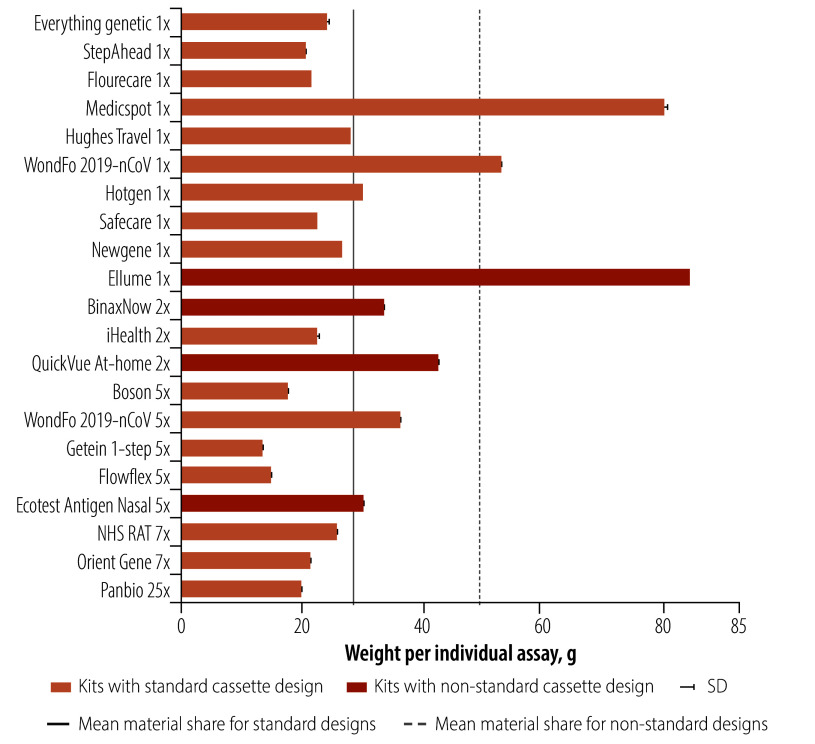
Total weight of lateral flow tests by brand

The total weight of the kits, divided by the number of individual tests, ranged from 13.7 g to 84.6 g per individual test, with a mean of 32.2 g (SD: 19.1; Fig. 1). The heaviest kit, Medicspot, is a single Flowflex test that has been repackaged in a larger heavier cardboard box. The kit includes a small card containing information on how to register the test to obtain a travel certificate.

CoV: coronavirus; NHS RAT: National Health Service rapid antigen test.

Note: further information about the tests is available in Table 1.

The tests included single-test kits and multitest kits containing two, five, seven and 25 tests. The Wondfo kit had both single-test and two-test kits. Comparing the weight of a single Flowflex (inside the Medicspot packaging) with the Flowflex five-test kit showed that the multitest kits required less overall material per test. The five-test pack was 46% (13.1/28.1) lighter per test than the one-test pack. Similarly, comparing the weight of the Wondfo one- and two-test kits showed 31% (16.4/53.3) less overall weight per test for the two-test kit. While an overall similar trend can be observed by comparing the average test weights of all single (39.4 g; SD: 24.5) and two-pack, (33.2 g; SD: 9.9), five-pack (20.9 g; SD: 10.8), seven-pack (26.0 g; SD: 4.4) and 25-pack (20.1 g; SD: not applicable) kits, the variations between the kits were too large for this trend to be statistically significant.

The four heaviest components on average in test kits were the outer packaging (mean: 11.5 g; SD: 13.0), cassette pack (mean: 7.7 g; SD: 4.2), instructions (mean: 7.0 g; SD: 5.9) and reagent pack (mean: 3.2 g; SD: 2.4). The other components (certification slip, biosafety or waste bag, swab pack, tube rack and control swabs) weighed substantially less. The outer packaging accounted for the largest weight variations in the total kit weights, largely because of the Medicspot kit, which is a Flowflex single-pack kit nested in a large outer packaging. When we excluded this kit as an outlier, the component with the largest variation was the instructions, followed by the cassette pack.

### Cassette designs and weights

**Fig. 2:**
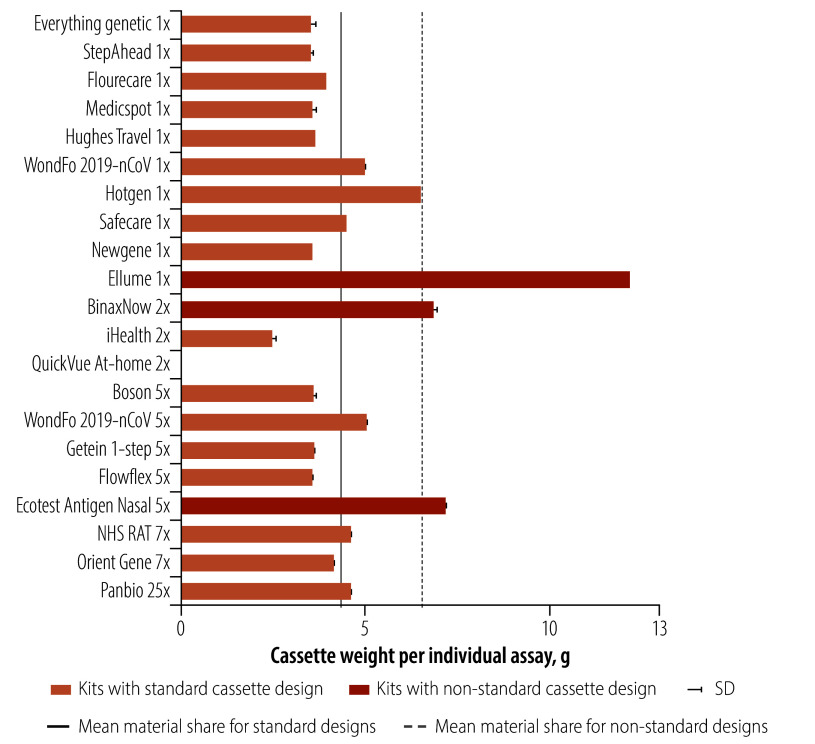
Weight of the hard plastic cassette casing component of tests by brand

CoV: coronavirus; NHS RAT: National Health Service rapid antigen test; SD: standard deviation.

Notes: some SDs are small and not distinguishable. Further information about the tests is available in Table 1.

Seventeen of the 21 COVID-19 lateral flow tests had a nitrocellulose strip in a shallow hard plastic cassette casing. The design was consistent across tests, usually about 2 cm wide, 6–9 cm long and 0.5 cm thick. Of these 17 designs, considering outer casing top (component G3.2) and outer casing bottom (G3.3) combined, we found a 20% (0.84/4.26) deviation in cassette weight, with an average of 4.1 g  (SD: 0.9; range: 2.5–6.5; Fig. 2). 

Four of the kits had a non-standard cassette design for encasing the nitrocellulose strip (Fig. 2). The QuickVue test did not use a test cassette at all. The extraction tube, already prefilled with reagent, was used to conduct the test: after taking the sample and mixing it in the tube, the swab is removed and the lateral flow test strip, which is stored on its own in a foil pouch without casing or desiccant, is dipped into the tube. The BinaxNow test used a cardboard cassette (no hard plastic) for the lateral flow test strip, and the reagent is dripped onto the swab with the sample inside the cardboard cassette. Otherwise, this test was packaged and set up like a cassette test. The Ellume test is a digital battery-powered COVID-19 test, which includes a large hard plastic casing weighing 12.2 g hollow and 22.0 g including its analyser printed circuit board, battery button and further internal elements. Additionally, we included the Ecotest test, available in Germany, which combines a swab, cassette and extraction tube into one pen-shaped device. The equivalent cassette element weighed 7.3 g.

### Swab designs and weights

**Fig. 3:**
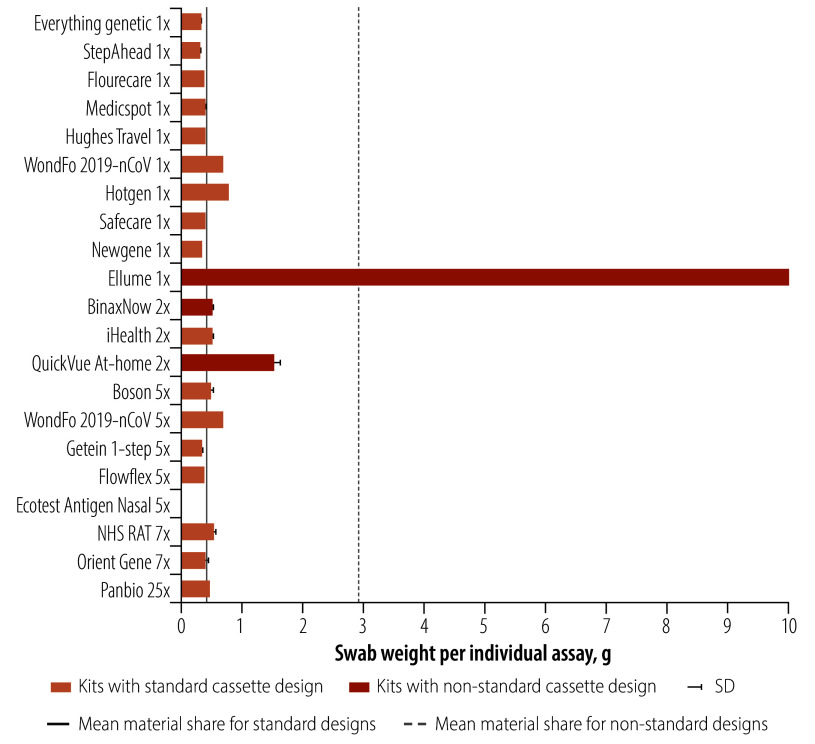
Weight of the swab component of tests by brand

CoV: coronavirus; NHS RAT: National Health Service rapid antigen test; SD: standard deviation.

Notes: some SDs are small and not distinguishable. Further information about the tests is available in Table 1.

Swabs are usually purchased from a small range of suppliers rather than manufactured alongside the test, hence this component has the lowest variations among the kits. The weights provided describe the swab only (component E.1). The average weight of swabs in the 16 standard design kits was 0.4 g (range: 0.3–0.7). The main difference between swabs was the length and thickness of the swab shaft, which is reflected in the overall weight (Fig. 3). Additionally, swab lengths had an effect on the overall packaging size, longer swabs required larger boxes and larger waste bags. We found large differences in the non-standard kits, with the Ellume swab and QuickVue swab weighing 9.8 g and 1.5 g, respectively. All other swabs weighted less than 1g. A photograph of all swabs is available in the online repository.^24^

### Extraction tube designs and weights

**Fig. 4:**
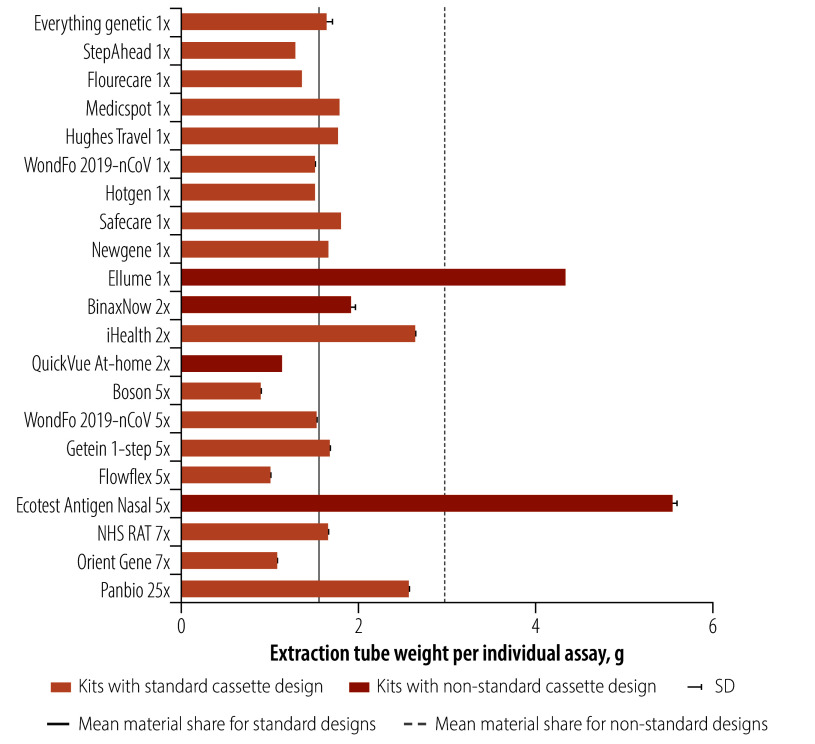
Weight of extraction tube component of tests by brand

CoV: coronavirus; NHS RAT: National Health Service rapid antigen test; SD: standard deviation.

Notes: some SDs are small and not distinguishable. Further information about the tests is available in Table 1.

The biggest conceptual difference between test kits was in the packaging of the reagent and the design of the extraction tube. Extraction tubes were closed either with foil lids or screw-on caps, which had little effect on the component weights overall. Most tests used the extraction tube to also store the reagent liquid. The Boson test, which used a separate reagent blister and extraction tube, and the Panbio test, which provided 9 mL of reagent in a reagent bottle for 25 tests were the exceptions (online repository).^24^ The weights of the extraction tubes (for the empty tube only) by test type are shown in Fig. 4. The heaviest reagent tube design weighed 5.6 g (Ecotest) and the lightest weighed 1.3 g (StepAhead). The average weight of reagent tubes in the standard test kits was 1.6 g, notably different from the reagent tubes of the four non-standard kits which averaged 3.3 g. In the standard design kits, the heaviest reagent and extraction tube solution used a tube, a screw-on cap and an additional screw-on cap or plug to secure the liquid.

### Packaging proportions

**Fig. 5:**
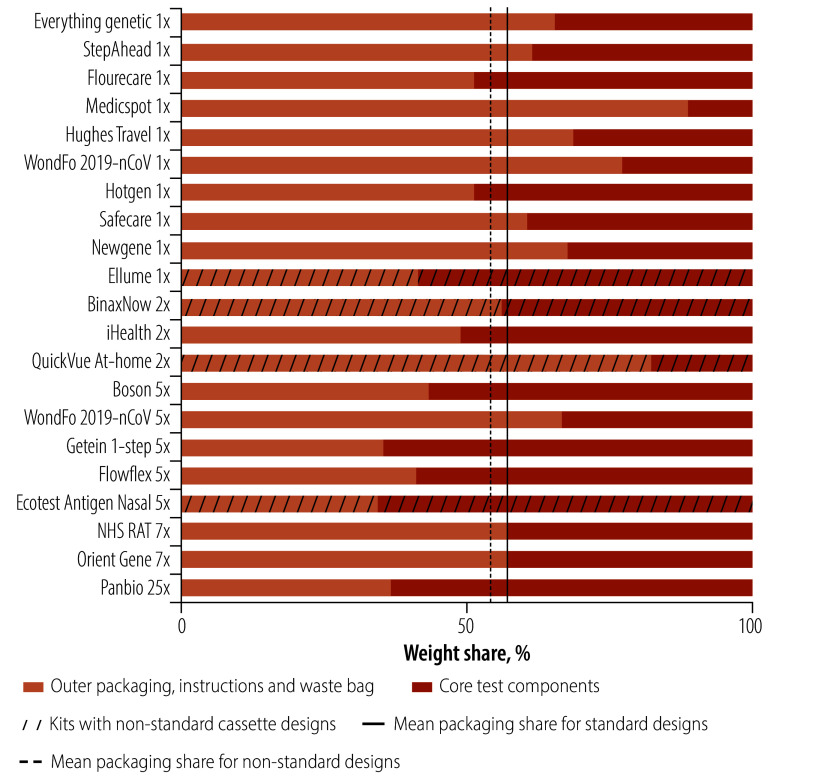
Weight proportions of packaging-related materials and core test components

Packaging design and decisions affect the overall weight of the kits considerably and thus their environmental footprint. All packaging-related components (components A to D and H to I) were grouped together and their weights compared to the core test components (components E to G). We observed large variations in the distribution of packaging weight across different components (Fig. 5). In 14 of the kits, all related packaging represented a larger weight proportion than the test cassette and other ancillary parts necessary to conduct the assay. Packaging made up on average 57% of the whole test kit weight, with large variations between 34% and 89%. The QuickVue test was the lightest test itself, but it had one of the highest total weights due to the amount of packaging materials, including a large plastic tray. Among the standard kits, subcomponents – cassette housings, swabs and tubes – had significant variations in weight with coefficient of variations of 20%, 33% and 25%, respectively, for the same test function.

CoV: coronavirus; NHS RAT: National Health Service rapid antigen test.

Note: further information about the tests is available in Table 1.

Peripheral to packaging, but also of note, were the different weights of the instruction booklets. Instructions varied in length between brands. This difference depended partially on the number of languages required – a test sold in the United Kingdom generally only includes English instructions, a test sold in the USA also usually includes a Spanish instruction booklet. WHO emergency use listing tests usually came with English instructions, although some manufacturers provided additional translations in a variety of languages.

**Fig. 6:**
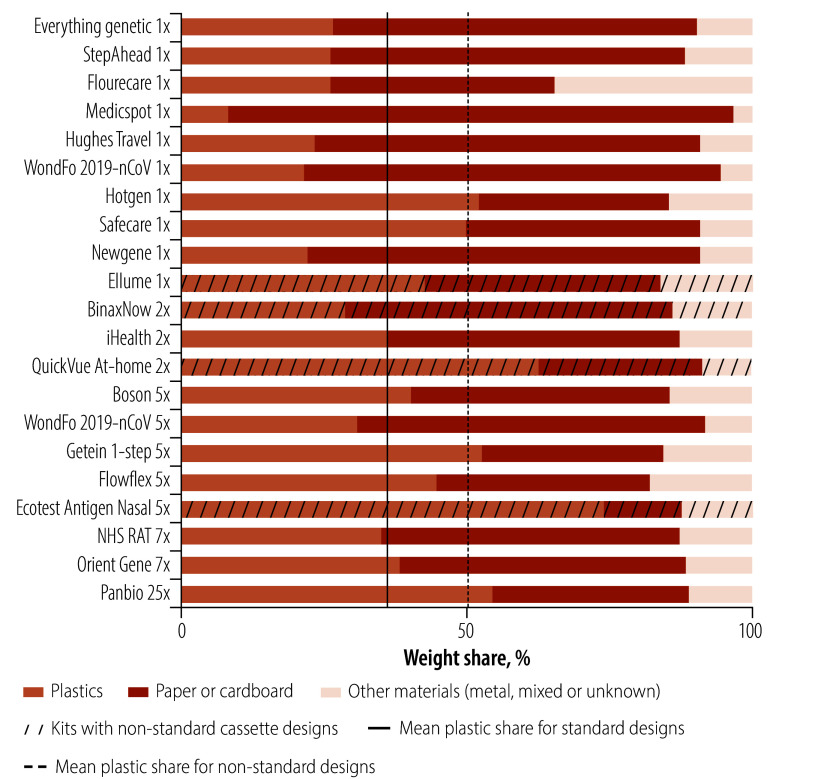
Weight proportions of plastics, paper or cardboard and other materials

We also analysed the type of materials and their weight distribution (Fig. 6). In the standard kits, on average, plastics made up 36% of the total weight of the kit, while paper and cardboard accounted for 52%. Conversely, in the non-standard kits, which had newer cassette designs, on average plastics constituted 52% of the total weight, with paper and cardboard representing 35%. 

CoV: coronavirus; NHS RAT: National Health Service rapid antigen test.

Note: further information about the tests is available in Table 1.

## Discussion

We compared the component weights of a range of lateral flow assays commercially available for at-home COVID-19 testing in France, Germany, the United Kingdom and USA to highlight differences in material use, even among almost identically shaped cassette tests, swabs or reagent tubes. This quantitative analysis offers insight into typical industry practices for the design and weight of lateral flow components.

Packaging was also found to vary significantly from 34% of the total kit weight up to 89% of the kit weight. The non-standard designs were on average 1.8 times heavier than the standard designs with the cassette subcomponent contributing to about 60% of the weight on average.

The wide range of component and packaging weights indicates inconsistency in material use. Excessive packaging contributes to higher levels of waste, increasing the burden on waste management systems and landfills. At the same time, heavier and bulkier packaging increases transportation costs and energy consumption (more fuel used) leading to more greenhouse gas emissions. Lighter, more compact kits and standardized packaging could decrease resource extraction, lower energy use in production, lower transport costs and reduce emissions associated with distribution. These results illustrate that there is scope for much lighter cassette designs and that, without policy guidance, innovative designs can go either way in terms of material usage and environmental sustainability.

Packaging is an area where waste reduction can be easily achieved. Our findings suggest that reducing packaging mass has not been a consistent priority in COVID-19 test design. Emphasizing eco-friendly packaging as a design criterion could drive the industry towards more sustainable practices without compromising the test’s functionality. For example, our findings show that bulk solutions have lower material usage per test. However, bulk kits have limited accessibility for low-income users and they are most likely to be used in clinical settings. Eliminating waste bags and reducing the size of paper instructions are other options to reduce the overall weight of kits and the waste produced. Reducing the overall packaging size allows more parts to fit within the same transport space (for example, a shipping container, truck or pallet). This efficiency reduces the per-unit transport footprint, leading to potential cost savings and environmental benefits such as reduced fuel consumption and emissions. Of interest, Abbott (manufacturer of the BinaxNow and Panbio tests) addressed packaging in an article on their website, announcing they had reduced packaging weight and volume for both test lines by removing internal plastic trays and reducing package size in line with their 2030 sustainability plan.^25^

Market average weights could form the basis for quantitative recommendations for the sustainability sections of target product profiles, which have until now been qualitative and unambitious. Although the overall weight of lateral flow kits rarely exceeds 30 g, these devices are produced in billions every year out of new plastics, adding a considerable amount of medical waste. Furthermore, production is expected to increase yearly with new types of tests appearing on the market, such as nucleic acid-based lateral flow assays.^26^^,^^27^ The results of our study can contribute to an evidence base for recommendations for minimum and optimal weight thresholds for such tests. This evidence supports the idea of setting limits in target product profiles, such as component weights not exceeding the market average of a specified amount, or guidelines specifying, for example, that kit packaging weight (per test for multitest kits) should not exceed a certain percentage of the total kit weight. These measures could encourage industry towards more efficient, minimalistic designs that would reduce excess material and support the transition towards more sustainable materials and the abandonment of plastics.

Traditional petrochemical-based plastics, although reliable for strength and durability, generate a substantial amount of waste and carbon dioxide emissions. Alternative solutions are emerging: card-based devices are already in use, illustrated by the BinaxNow test in our study. Bioplastics,^15^^,^^28^^,^^29^ cellulose^30^ and recycled materials^31^ have also been proposed as materials with lower environmental impact. Some brands, particularly those producing pregnancy and reproduction tests, are opting for reusable components^32^ and simple strips,^33^^,^^34^ or are promoting recycling through take-back schemes.^35^ However, none of these products is yet in high-volume production or destined for the global health market. More research is needed to understand the technical, financial and regulatory effects of transitioning to alternative formats.

To conclude, more than 2 billion lateral flow tests are produced every year, adding tens of thousands of tonnes of used materials to the global volume of medical waste. Our study establishes a baseline for material usage, underscoring opportunities to reduce material consumption, especially plastics, in test components. Our study contributes to a wider evidence base that can be used by policy-makers to refine environmental criteria in the REASSURED guidance^20^ to limit material use and reduce reliance on new petrochemical plastics in point-of-care diagnostics. The findings can also inform optimum target weights for individual components or entire tests in future target product profiles, offering concrete targets to address health care’s environmental impact. Our findings also highlight an urgent need to explore alternative packaging designs. By seeking more efficient and eco-friendly designs and materials, manufacturers can help mitigate environmental impact while maintaining product integrity and performance.
